# Cracking the combination: Gut hormones for the treatment of obesity and diabetes

**DOI:** 10.1111/jne.12664

**Published:** 2019-01-02

**Authors:** Kleopatra Alexiadou, Oluwaseun Anyiam, Tricia Tan

**Affiliations:** ^1^ Section of Investigative Medicine Imperial College London London UK

## Abstract

Obesity and type 2 diabetes are a veritable global pandemic. There is an imperative to develop new therapies for these conditions that can be delivered at scale to patients, which deliver effective and titratable weight loss, amelioration of diabetes, prevention of diabetic complications and improvements in cardiovascular health. Although agents based on glucagon‐like peptide‐1 (GLP‐1) are now in routine use for diabetes and obesity, the limited efficacy of such drugs means that newer agents are required. By combining the effects of GLP‐1 with other gut and metabolic hormones such as glucagon (GCG), oxyntomodulin, glucose‐dependent insulinotropic peptide (GIP) and peptide YY (PYY), we may obtain improved weight loss, increased energy expenditure and improved metabolic profiles. Drugs based on dual agonism of GLP1R/GCGR and GLP1R/GIPR are being actively developed in clinical trials. Triple agonism, for example with GLPR1/GCGR/GIPR unimolecular agonists or using GLP‐1/oxyntomodulin/PYY, is also being explored. Multi‐agonist drugs seem set to deliver the next generation of therapies for diabetes and obesity soon.

## 1 THE PROBLEM OF OBESITY AND DIABETES

The prevalence of overweight and obesity has dramatically increased over the last decades posing a significant health problem with associated complications and major socioeconomic impact. According to the WHO, worldwide obesity has nearly tripled since 1975. In 2016, more than 1.9 billion adults were overweight of which 650 million were obese. These numbers correspond respectively to 39% and 13% of the adult population.^1^ Obesity drives the pathogenesis of other diseases such as type 2 diabetes, cardiovascular disease, related musculoskeletal disorders such as osteoarthritis and some cancers (endometrial, breast, ovarian, prostate, liver, gallbladder, kidney and colon).^2^ Obesity is therefore responsible for a great deal of mortality throughout the world.^3^ The aetiology of obesity lies fundamentally in an imbalance of energy homeostasis from the interaction of three factors: (a) increased energy intake through the supply of unlimited quantities of energy dense food and drink which are tuned to appeal to our appetites; (b) decreased energy expenditure as result of modern lifestyles (a more sedentary environment, contemporary modes of transportation, urbanization, reduction in sleep quality); (c) genetic and ethnic predispositions to obesity.

Although bariatric surgery is clearly an effective treatment for obesity with long‐term follow‐up data proving its efficacy and success in improving lifespan, enforcing a remission of diabetes in some patients and ameliorating other obesity‐associated co‐morbidities,^4^ it can never be the sole solution. Firstly, given the numbers of people with obesity, bariatric surgery can never be implemented at the scale necessary without immense expense for healthcare systems. Secondly, bariatric surgery can be unpredictable in its effects, leading to varying levels of weight loss and diabetes remission from standardized “one size” procedures which do not necessarily fit all. Thirdly, complications such as post‐prandial hypoglycaemia and nutritional deficiencies cause long‐term morbidity and require long‐term follow‐up. Furthermore, a large part of the morbidity associated with overweight and obesity is found in overweight people,^3^ and the magnitudes of weight loss from bariatric surgery may be inappropriate for this population. Therefore, there is an imperative to develop new therapies which deliver key benefits such as titratable and effective weight loss, amelioration of diabetes, prevention of diabetic complications and improvements in cardiovascular health. This review will look at the latest developments in the sphere of gut hormone treatments for obesity and diabetes and consider whether combination gut hormone therapies may prove to be the next big thing in obesity treatment.

## 2 GLP‐1 ACTION, CLINICAL APPLICATIONS AND LIMITATIONS

Glucagon‐like peptide‐1 (GLP‐1) has been the forerunner of the gut hormone‐based therapies. GLP‐1_7‐37_ and GLP‐1_7‐36amide_ are secreted from the neuroendocrine L‐cells of the small intestine. GLP‐1 together with glucose‐dependent insulinotropic peptide (GIP) are responsible for the incretin effect, ie, augmented insulinotropy in response to an ingested glucose load as opposed to an isoglycaemic intravenous glucose challenge.^5^ The incretin effect is reduced in type 2 diabetes.^6^ GLP‐1 is derived from the post‐translational processing of proglucagon in L‐cells and some brainstem neurones by prohormone convertase 1 (PC1). Classically, proglucagon processing by PC2 in pancreatic α cells releases glucagon itself,^7^, ^8^ but a subset of α cells has been shown to process proglucagon to GLP‐1.^9^ Proglucagon is additionally processed in L‐cells to the products glicentin, oxyntomodulin (identical to glucagon with a C‐terminal octapeptide extension) and GLP‐2. After secretion, GLP‐1 has a short half‐life of 1‐2 minutes as it is rapidly degraded and inactivated by the endopeptidase dipeptidyl peptidase‐4 (DPP‐IV), resulting in the formation of the inactive metabolite GLP‐1_9‐37_ and GLP‐1_9‐36amide_. GLP‐1 exerts its effects via the GLP‐1 receptor (GLP1R) which belongs to the family of G protein‐coupled receptors. GLP1R is abundantly expressed in the pancreas, gut and central nervous system but also in the heart, lungs, vasculature and peripheral nervous system.^10^ As noted above, GLP1R activation on β cells causes enhanced glucose dependent insulin secretion^11^; at the same time it suppresses glucagon secretion from α cells. The effects of GLP‐1 on β cells extend beyond the insulinotropic effect on glucose homeostasis including inhibition of β cell apoptosis, induction of their proliferation and expansion of their mass in rodents.^9^ GLP1R agonism reduces food intake in animals^12^, ^13^ and humans^14^, ^15^, ^16^ acting on regions of the hypothalamus and hindbrain as evidenced in animal studies.^17^, ^18^ GLP‐1 also acts in the gastrointestinal tract through inhibition of gastric secretion and deceleration of gastric emptying^19^ attenuating the postprandial rise in glucose levels. It also reduces hepatic steatosis, liver inflammation and hepatocyte injury; these effects could be either direct^20^ or indirect through weight loss.^9^, ^21^ Amongst the multifaceted mechanisms of action of GLP‐1 is also its ability to activate invariant natural killer cells (i‐NKT) which triggers the production of fibroblast growth factor 21 leading to weight loss in mice.^22^ GLP‐1 may also possess neurotropic effects improving learning in rats and exerting neuroprotective effects.^23^, ^24^ The presence of GLP‐1 receptors in the heart suggests a physiological role of GLP‐1 in cardiac function.^25^ Mice lacking the GLP1R have reduced resting heart rate, increased left ventricular end‐diastolic pressure and increased left ventricular wall thickness.^26^ GLP1R agonists have a characteristic positive chronotropic effect which is reduced but not completely abrogated in mice with cardiomyocyte‐selective knockouts for GLP1R, suggesting that the effect is partially mediated by a direct effect on cardiomyocytes and partially via the autonomic nervous system.^27^

Such diversity of actions of GLP‐1 has led to the development of GLP‐1 based therapies for improving glycaemia in type 2 diabetes, for weight loss, and more recently for improving cardiovascular outcomes in patients with diabetes and established cardiovascular disease. The first GLP1R agonist approved for clinical use was exenatide (synthetic exendin‐4), a peptide originally isolated from *Heloderma suspectum* lizard venom by John Eng in 1992.^28^ Pivotal studies in patients with type 2 diabetes led to the approval of twice daily exenatide which was the first GLP1R agonist in the market in 2005. Since then, other GLP1R agonists have been marketed including Lixisenatide, Liraglutide, Dulaglutide, Albiglutide and Semaglutide. Exenatide LAR, Dulaglutide and Semaglutide are notable in that they are long‐lasting preparations, enabling effective treatment of type 2 diabetes with one injection a week, an attractive proposition for patients.^29^ One of the key properties of GLP1R agonists that sets them apart from other diabetes treatments is the weight loss associated with treatment, which varies in trials from 1.01 to 1.62 kg mean weight loss at the doses utilised for diabetes.^30^ As a result, the wider use of GLP1R agonists in overweight/obesity has been explored, and Liraglutide was the first GLP1R agonist to be approved for the treatment of obesity as a once daily injection in 2014, based on studies utilising higher doses of 3 mg daily in overweight and obese patients with and without diabetes. At 3 mg daily, Liraglutide can reduce weight by a mean of 8%.^31^ The GLP‐1 analogues Liraglutide,^32^ Semaglutide,^33^ Exenatide LAR,^34^ Albiglutide,^35^ and Dulaglutide^36^ have recently been shown to reduce cardiovascular events (notably non‐fatal myocardial infarctions) in diabetic patients at high risk for cardiovascular disease, an effect that has been attributed to reduction in cardiovascular inflammation, although the exact mechanisms remain obscure.^29^

The efficacy of GLP1R agonists is partially limited by the adverse effects which are mainly gastrointestinal in nature (nausea, vomiting, loose stools or constipation), although these adverse effects are subject to tachyphylaxis and can be mostly avoided with a slow up‐titration in dose. Another limitation is that GLP1R agonist treatment does not significantly increase energy expenditure.^37^ Patients can vary widely in their weight loss responses to Liraglutide 3 mg: despite the abovementioned mean weight loss, the SCALE trial also demonstrated that 37% of patients given liraglutide lost less than the minimally acceptable weight loss of 5% (some even gained weight).^31^ Even for those that respond to treatment, a mean weight loss of 8% is not enough to address higher grades of obesity, and is not competitive with the typical weight loss from bariatric surgery of 20% or so.^38^ Newer GLP1R agonists such as Semaglutide possess better efficacy in terms of glycaemic improvement^39^ and weight loss (with mean reductions in weight of 6%‐14%).^40^ Nevertheless, to achieve better and titratable outcomes, for example 15%‐20% weight loss, it will be necessary to exploit the power of combination therapy with other gut hormones.

## 3 WHAT OTHER HORMONE ACTIONS CAN BE COMBINED WITH GLP‐1's ACTIONS?

### 3.1 Glucagon

Glucagon is a 29‐amino acid peptide hormone that is produced by the α cells of the pancreatic islets^41^ as an alternative product of post‐translational proglucagon processing by PC2. Glucagon activates the G‐protein coupled glucagon receptor (GCGR) which is expressed most abundantly in the liver and kidney but to a lesser extent in cardiac, adrenal, gut and adipose tissues.^42^ Historically, glucagon was characterised as a hyperglycaemic hormone, increasing hepatic glucose production via glycogenolysis and gluconeogenesis.^43^ Its secretion is stimulated by low blood glucose levels or fasting, and glucagon classically acts as a counter‐regulatory hormone to insulin. In addition, glucagon stimulates lipolysis from adipose tissue^44^, ^45^; decreases muscle protein synthesis^46^; increases hepatic amino acid uptake, amino acid catabolism, and ureagenesis^47^; and acts as an insulinotropic hormone.^48^ Further studies of glucagon have demonstrated a range of other metabolic effects. It has been consistently demonstrated to increase energy expenditure through a mechanism that appears to be independent of brown adipose tissue.^49^, ^50^, ^51^ Furthermore, glucagon directly affects satiety and its infusion has been shown to reduce food intake,^52^ seemingly via both central and peripheral pathways.^53^, ^54^ Two clinical studies conducted by our group have demonstrated the potential beneficial effects of GLP‐1/glucagon combination in humans. Ten non‐diabetic overweight/obese individuals were co‐infused with glucagon and GLP‐1 for 45 minutes at doses of 50 ng/kg/min and 0.8 pmol/kg/min respectively.^55^ On different occasions, single hormone infusions were also administered (at the same doses), as well as a placebo infusion. Resting energy expenditure—as determined by indirect calorimetry—increased significantly in both the glucagon and combined infusion groups, but not in the GLP‐1 infusion group. Whilst the glucagon infusion caused a rise in plasma glucose as expected, this rise was blunted in the combined infusion, owing to a significant synergistic effect between GLP‐1 and glucagon on insulin secretion.^55^ A subsequent study by Cegla et al^56^ confirmed that the addition of GLP‐1 to glucagon infusion protected from glucagon induced hyperglycaemia, and that co‐infusion of GLP‐1 and glucagon at sub‐anorectic doses led to a significant and synergistic reduction in food intake of 13%. Hence, the GLP‐1/glucagon combination possesses three highly favourable features over and above the individual hormones, ie, (a) synergistic reduction in food intake; (b) an increase in energy expenditure that would counteract any tendency to reduce resting energy expenditure with weight loss; (c) gluconeutrality, with the possibility of improved glycaemia in the long term with weight loss.

### 3.2 Oxyntomodulin

Oxyntomodulin is co‐secreted by the L cells of the small intestine with GLP‐1. As noted above, it is an additional posttranslational product of proglucagon in the gut. In addition to the glucagon sequence, oxyntomodulin has a C‐terminal eight amino acid octapeptide.^57^ Oxyntomodulin is an agonist for both GLP1R and GCGR (albeit with less affinity than the cognate peptide for each receptor), making it the prototypical unimolecular GLP‐1/glucagon dual agonist.^58^ Indeed, studies have shown that the effect of oxyntomodulin via the GLP1R is anorectic whereas the observed increase in energy expenditure is mediated via the glucagon receptor.^59^, ^60^, ^61^ Pocai et al characterised the preclinical effects of a GLP1R/GCGR dual agonist based on oxyntomodulin. They demonstrated a significant reduction in body weight, food intake and fat mass of a greater magnitude compared to a pure GLP1R agonist. In addition, they again demonstrated improvements in fasting glucose and glycaemic profiles post‐glucose tolerance test which were comparable to the GLP1R agonist.^58^ The effects of a short IV infusion of the combination of GLP‐1 and OXM on food intake were examined a study performed by Field et al^62^ in overweight subjects, where a synergistic effect from combining GLP‐1 and OXM resulted in a 42% reduction in energy intake.

### 3.3 Glucose‐dependent insulinotropic peptide

Glucose‐dependent insulinotropic peptide is a peptide secreted by the neuroendocrine K cells of the small intestine; its principal physiological roles, mediated by the GIP receptor GIPR, are as an incretin (with GLP‐1), potentiation of glucagon secretion, and regulation of adipogenesis, notably increasing fat deposition in adipose tissue. The insulinotropic properties of GIP make it an attractive prospect for treating type 2 diabetes. Although the beneficial effects of GIPR agonism appear to be attenuated in the hyperglycaemic conditions seen in patients with type 2 diabetes,^63^ even when administered at supraphysiological levels,^64^, ^65^ the impaired insulinotropic effect of GIP seems to be fully recoverable following a period of normalised plasma glucose levels.^66^ This suggests a role for co‐agonism with GLP‐1, utilising its glucose lowering effects to induce improvement in glycaemia that is enough to enable GIP to exert its insulinotropic effects and synergistically improve glucose levels further. The initial preclinical study by Irwin et al^67^ demonstrated improvement in glucose tolerance, as well as in food intake and weight reduction with a combination of the GLP1R agonist exendin‐4 and the GIPR agonist N‐AcGIP. These findings were corroborated in a subsequent preclinical study which demonstrated a synergistic effect of the combination on improved glucose levels, reduced food intake and weight loss.^68^ The data from clinical studies into the acute effects of combined GLP‐1/GIP in patients with type 2 diabetes have not been as promising however. In a study by Daousi et al,^69^ GLP‐1 and GIP were infused individually and in combination into six healthy lean participants and six overweight diabetic participants. Whilst the combined infusion led to a greater potentiation of insulin secretion in the lean group, this effect was not observed in the overweight diabetic group where insulin levels matched those from the GLP‐1 only infusion. The excursion of glucose in response to intravenous insulin, as measured by the AUC for glucose, was similar between GLP‐1 and GLP‐1/GIP, as was the food intake during an ad libitum meal test. There was a reduction in resting energy expenditure with GIP which was not replicated during combined infusion in both groups. The lack of synergistic effect in type 2 diabetic patients is consistent with the established evidence that GIP‐mediated insulin secretion is less effective in the hyperglycaemic state. Nevertheless, GLP‐1/GIP based dual agonists have continued into clinical development (see below). Other groups have explored GIPR antagonism, which might be therapeutically attractive as a means of suppressing the hyperglucagonaemia and hence hyperglycaemia of type 2 diabetes, as well as reducing fat deposition.^70^ GIPR antagonism with (Pro^3^)GIP was shown to ameliorate weight gain, insulin resistance and normalise glucose tolerance in high‐fat diet fed mice.^71^ Some exploratory work looking at the physiological effects of GIPR antagonism in human volunteers has recently been published which demonstrates that the antagonist GIP_3‐30amide_ is capable of suppressing the incretin effect of GIP,^72^ but it is too early to tell yet whether GIPR antagonism may be a valid therapeutic strategy.

### 3.4 Peptide YY

Peptide YY (PYY) is another peptide hormone secreted by the L‐cells of the intestine in response to eating.^73^ The PYY_3‐36_ peptide, which is derived from the full‐length PYY_1‐36_ peptide by DPP‐IV processing, binds to the neuropeptide Y2 (Y2R) and Y5 (Y5R) receptors, and has a well‐characterised appetite‐suppressive effect which is importantly preserved in obesity.^74^ Neary et al studied the coadministration of PYY_3‐36_ with GLP‐1_7‐36amide_ in 10 healthy volunteers and found that it was associated with a 27% reduction in energy intake from a buffet meal. The combination was more effective in inhibiting appetite than either peptide alone.^75^ This result was replicated in a later study by De Silva et al^76^ where the combination of PYY_3‐36_ and GLP‐1_7‐36amide_ resulted in a reduction in food intake which was similar to the summed effects of the single hormones (PYY_3‐36_ or GLP‐1_7‐36amide_), and this was reflected by a reduced activation, as assessed by BOLD fMRI, of areas of the brain implicated in appetite and interest in food. Similarly, Schmidt et al^77^ in 2014 showed that the co‐infusion of GLP‐1 and PYY_3‐36_ reduced energy intake compared with placebo and more than the sum of the individual infusions, demonstrating a synergistic effect. Therefore, the combination of GLP‐1 and PYY_3‐36_ bears some promise of augmented weight loss compared to GLP‐1 alone.

## 4 DEVELOPMENT OF DUAL AGONISTS

### 4.1 GLP1R/GCGR dual agonists

As noted above, there are positive therapeutic effects of GLP‐1/glucagon combinations in terms of increasing energy expenditure and reducing food intake; GLP‐1 can counterbalance the hyperglycaemia induced by glucagon. As a result, there has been considerable interest in developing GLP‐1/glucagon dual agonists. In early work from 2009, Day et al^78^ developed a range of unimolecular co‐agonists and tested their binding affinities to both glucagon and GLP‐1 receptors. Following this, they tested two of the novel peptides in mice and showed reductions in body weight, fat mass and most pertinently, reduction in blood glucose. In addition, despite the lack of hyperglycaemia induced by the co‐agonist, the glucagon‐associated effects of increased energy expenditure and improved lipid profile were preserved.

A recently published Phase 2A, randomized, double‐blind, placebo controlled trial assessed the efficacy, safety and tolerability of MEDI0382 (AstraZeneca/Medimmune), a GLP1R/GCGR dual agonist in overweight and obese patients with type 2 diabetes.^79^ The volunteers given MEDI0382 once a day for 41 days were shown to have a better glucose tolerance in response to a mixed meal test compared to placebo, as well as a reduction in body weight: the mean fall in body weight between baseline and day 41 was 3.84 kg compared to 1.70 kg for placebo. HbA1c fell by 0.9% with MEDI0382 compared to 0.6% for placebo. Twenty patients experienced treatment‐emergent adverse events (reduced appetite, vomiting, headache) with MEDI0382, compared to 15 for placebo.^79^ Exploratory analyses presented in the American Diabetes Association 2018 meeting also suggest that there was a significant higher relative reduction in liver fat content when patients with fatty liver disease given MEDI0382 vs placebo, a potentially important effect.^80^

Another GLP1R/GCGR dual agonist, SAR425899 (Sanofi, Frankfurt, Germany) has recently been evaluated in single‐ascending dose and multiple‐ascending dose Phase 1 trials when given once a day over 28 days.^81^ At the highest maintenance doses tested, there was a reduction of HbA1c by 0.54%‐0.59% when given to overweight/obese diabetic patients, and mean weight losses of 2.37‐5.46 kg over the 28 days. SAR425899 was generally well tolerated, with treatment‐emergent adverse effects of reduced appetite and nausea. It should be noted that two patients in the study were withdrawn due to increases in serum lipase activity. These promising trial results therefore suggest that GLP1R/GCGR dual agonism is generally safe and efficacious, although head‐to‐head comparisons with GLP1R agonists will be required to evaluate the comparative advantages of the GLP1R/GCGR dual agonists.

### 4.2 GLP1R/GIPR dual agonists

Notwithstanding the equivocal evidence for benefit from GLP‐1/GIP co‐infusion studies, unimolecular GLP1R/GIPR dual agonists have been developed with promising pre‐clinical results (enhanced weight loss, improved glycaemia, reduced hepatosteatosis) in animal models.^82^ One recent study has reported the Phase 2A clinical trial results of one such dual agonist, NNC0090‐2746, which possesses balanced affinities for the GIPR and the GLP1R.^83^ A 1.8 mg once‐daily dose was administered to 96 overweight/obese patients with type 2 diabetes. There was a significant reduction in HbA1c of 0.63% and 0.96% at 8 and 12 weeks respectively when compared to placebo. In addition, body weight was significantly reduced by 1.8% after 8 weeks (when compared to placebo), although the reduction in body weight was not significant at 12 weeks. Post‐hoc analysis found that the best weight reduction and improvement in HbA1c was demonstrated in the group of patients with HbA1c <8.5%. This finding was again in keeping with the previous observations that GIP is less effective in more hyperglycaemic conditions.

More impressive, however, are the data from Frias et al^84^ who studied the effects of LY3298176, a once‐weekly GLP1R/GIPR dual agonist, in a Phase 2 trial in type 2 diabetic patients. LY3298176 differs from NNC0090‐2746 in that it possesses greater affinity for GIPR vs GLP1R. LY3298176, when given for 26 weeks, delivered mean reductions in HbA1c ranging from 1.06% to 1.94% in comparison to a group taking Dulaglutide, who had a mean reduction of HbA1c of 1.21%. Even more impressively, the LY3298176‐treated patients had mean weight reductions ranging between 0.9 and 11.3 kg, compared to 2.7 kg for Dulaglutide. Surprisingly, however, there was a 30% non‐response rate (ie, weight loss <5% of baseline) even when given the higher doses of the drug. LY3298176 was associated with nausea, diarrhoea and vomiting in up to 60% of the patients given the highest dose (35% for Dulaglutide). Nevertheless, these early results do suggest that LY3298176 possesses enhanced efficacy compared to a benchmark GLP‐1 analogue in terms of weight loss and improvements in glycaemia, and augurs well for this class of dual agonists.

### 4.3 GLP1R/Y2R dual agonism

Peptide YY analogues are currently under development for the treatment of obesity (ClinicalTrials.Org NCT01515319 and 85). Novo Nordisk has a peptide YY analogue (PYY‐1562 or NN‐9748) in Phase I trials; this is likely to be combined with GLP1R agonists such as Semaglutide to achieve enhanced effects on food intake suppression,^86^ following on from the proof‐of‐concept studies noted above.

## 5 TRIPLE AGONISM—MORE IS BETTER?

### 5.1 GLP1R/GIPR/GCGR triple agonism

Complex unimolecular triple agonists combining GLP‐1/GIP/glucagon activity such as MAR423, which are based on modification of a pre‐existing GLP1R/GIPR dual agonist to incorporate GCGR agonist activity have been designed by Richard DiMarchi and Matthias Tschöp,^87^ and pre‐clinical studies again suggest promising metabolic benefits in animal models of obesity.^88^, ^89^ At the same time, Hanmi Pharmaceutical has developed an independent triple agonist, HM15211, which also shows promising pre‐clinical benefits in a model of non‐alcoholic steatohepatitis.^90^ At present, no clinical trial or clinical study results are available for this class of triple agonists.

### 5.2 GLP‐1, Oxyntomodulin & PYY triple agonism—replicating bariatric surgery

As noted, Roux‐en‐Y Gastric Bypass (RYGB) surgery has been proven in multiple studies to be an effective treatment for obesity and diabetes.^4^ Dramatic changes in gut hormones, namely the rise in postprandial levels of GLP‐1, OXM and PYY even days after the operation, account for the improvement in glycaemic control which can reach diabetes remission as well as for the weight loss and other beneficial metabolic effects such as enhancement of insulin sensitivity and improved lipid profile.^91^, ^92^ We asked whether it might be possible to replicate the benefits of RYGB using a triple hormone infusion of GLP‐1, oxyntomodulin & PYY (GOP for short). In a proof of concept study, we demonstrated that a subcutaneous infusion of GOP hormones in ten obese healthy subjects for 10.5 hours can replicate the post‐prandial gut hormone levels seen after RYGB.^93^ Importantly, the GOP infusion can induce a mean reduction of food intake by 32%. Additionally, glucose and insulin levels after lunch and dinner were significantly lower on the GOP infusion compared to placebo. Resting energy expenditure showed a non‐significant elevation from baseline on the GOP infusion. Finally, the GOP infusion induced nausea in a minority of participants, which settled within the first 4 hours without any vomiting. Continuing studies are underway to determine the effects of the GOP infusion when given for extended periods of time.

## 6 CONCLUSIONS

**Figure 1:**
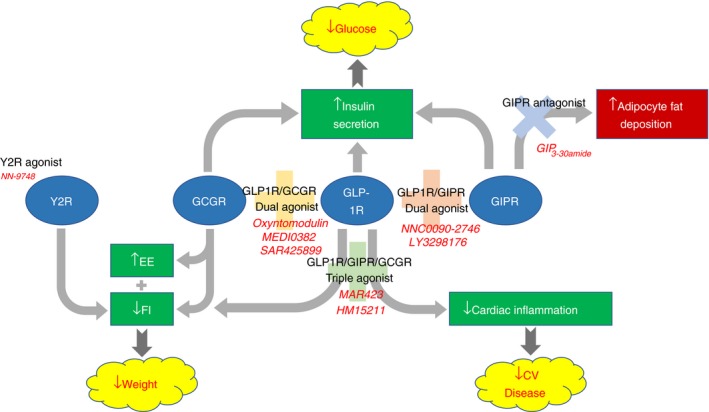
Complementary actions of gut hormone receptors combine to achieve desirable therapeutic outcomes

Decades of animal and human research in the field of gut hormones have produced effective and safe therapies for the treatment of diabetes and obesity with the GLP1R agonists now accepted as routine treatments for diabetes and obesity. Furthermore, we now have clinical evidence for the cardiovascular benefits of GLP1R agonists. To go beyond the modest effects of GLP1R agonism, we now need to understand how to combine the benefits of GLP‐1 with the complementary properties of its cousin gut hormones to achieve desired therapeutic goals (Figure 1). Dual and triple gut hormone receptor agonists are now placed to deliver the next generation of therapies for diabetes and obesity.
